# Machine learning prioritization identifies PANX1 as an inflammation-associated candidate regulator in lung adenocarcinoma

**DOI:** 10.3389/fgene.2026.1831053

**Published:** 2026-07-13

**Authors:** Ya Yang, Tingyuan Fan, Xiaye Miao, Chuanli Ren

**Affiliations:** 1 Department of Geriatrics, The Affiliated Huai’an Hospital of Xuzhou Medical University, Huai’an, China; 2 Department of Laboratory Medicine, The Yangzhou Clinical Medical College of Xuzhou Medical University, Yangzhou, Jiangsu, China; 3 Department of Laboratory Medicine, Northern Jiangsu People’s Hospital, Yangzhou, Jiangsu, China

**Keywords:** big data, inflammation, lung adenocarcinoma, machine learning, multi-omics, PANX1, prognostic biomarker, tumor microenvironment

## Abstract

**Background:**

Tumor-associated inflammation is an important contributor to cancer progression and therapeutic resistance. However, the molecular regulators linking inflammatory signaling with tumor biology in lung adenocarcinoma (LUAD) remain incompletely defined.

**Methods:**

We implemented an integrative multi-cohort and machine learning framework to identify inflammation-associated candidate regulators in LUAD. Immune-inflammatory genes derived from hallmark pathways were intersected with differentially expressed and prognosis-associated genes in TCGA-LUAD. Transcriptomic and clinical data from TCGA and 13 independent GEO cohorts were integrated for model development and validation. Multi-algorithm prioritization was applied to derive a consensus prognostic signature and nominate core candidates. Multi-omics analyses were conducted to characterize genomic alterations and immune associations. Functional relevance was examined using loss-of-function experiments in lung cancer cell lines.

**Results:**

We identified 52 inflammation-associated candidate genes and derived a 10-gene core signature with moderate prognostic performance across independent cohorts, while cohort and endpoint heterogeneity remained evident. Among these genes, PANX1 emerged as a focused candidate for further characterization based on an integrative assessment of model contribution, recurrence across analyses, and biological plausibility. Multi-omics analyses linked PANX1 expression to copy-number alterations, mutational context, and inferred immune microenvironment features. PANX1 was significantly upregulated in lung cancer cells, and its knockdown inhibited cell proliferation, reduced extracellular ATP release, and suppressed pro-inflammatory cytokine expression, including IL6, TNFA, and IL1B.

**Conclusion:**

By integrating large-scale multi-cohort analysis, machine learning prioritization, and experimental validation, this study identifies PANX1 as a potential inflammation-associated regulator in LUAD. These findings support a previously underrecognized association between PANX1 and inflammatory tumor biology and suggest its potential value as a biomarker candidate, while further mechanistic and translational validation is required.

## 1 Introduction

Lung adenocarcinoma (LUAD) is the most common histological subtype of lung cancer and remains a leading contributor to cancer-related mortality worldwide (Zhang Y. et al., 2023; Bray et al., 2024). Although targeted therapies and immunotherapies have improved clinical management, patient outcomes remain highly variable, and only a subset achieve durable therapeutic benefit (Ashrafi et al., 2022). This variability reflects, in part, the complexity of tumor-microenvironment interactions, where inflammatory signaling, immune infiltration, and tumor-intrinsic programs jointly shape disease progression and treatment response (Lim et al., 2023). A clearer identification of inflammation-associated molecular candidates that bridge tumor biology and the immune microenvironment is therefore important for improving risk stratification and for nominating molecules worthy of further functional investigation (Lin et al., 2024).

Tumor-associated inflammation is not merely a bystander phenomenon; it can promote tumor progression through cytokine networks, stress signaling, and metabolic rewiring, while also influencing immune surveillance and immune escape (Huang et al., 2022; Chi et al., 2025). In LUAD, inflammatory pathways such as TNFα–NF-κB and IL6–JAK–STAT3 are frequently activated and have been implicated in epithelial plasticity, therapy resistance, and immunosuppressive remodeling (Gong et al., 2021; Odarenko et al., 2023; Hu et al., 2024; Liu et al., 2026). However, translating these pathway-level insights into clinically useful biomarkers or targets is challenging. Individual studies often rely on single cohorts, limited gene panels, or single-algorithm feature selection, which can yield unstable signatures that fail to generalize across platforms and populations (Zhang P. et al., 2023; Ren et al., 2023). Moreover, many computational predictors remain weakly connected to functional evidence, limiting their translational interpretability.

Large-scale public datasets provide an opportunity to address these limitations, but extracting reproducible inflammation-associated candidate regulators requires careful integration and prioritization strategies (White et al., 2024). In particular, combining biologically grounded gene sets with multi-cohort validation and algorithmic consensus can reduce overfitting and improve reproducibility (Jin et al., 2022; Su et al., 2025). At the same time, integrating multi-omics layers-such as copy-number alterations, mutational context, and immune deconvolution-can help clarify whether candidate genes are embedded in coherent genomic and microenvironmental states (Zhang et al., 2024). Finally, basic experimental validation is essential to confirm whether computationally nominated candidates exert measurable phenotypic effects in LUAD cells.

In this study, we developed an integrative big-data and machine learning framework to identify inflammation-associated candidate regulators in LUAD. We curated immune-inflammatory genes from hallmark inflammatory pathways and intersected them with genes showing both differential expression and overall survival relevance in TCGA-LUAD, yielding a focused candidate set for downstream prioritization. We then applied multi-algorithm machine learning to reduce dimensionality and derived a 10-gene core signature, which was used to build a consensus prognostic model and validated across TCGA and 13 independent GEO cohorts. To move beyond prognostic association, we performed multi-omics analyses to characterize the genomic alteration landscape and inferred immune microenvironment correlates of key candidates, and we prioritized pannexin 1 (PANX1) for deeper investigation based on an integrative assessment rather than a single ranking metric.

We further conducted loss-of-function experiments in LUAD cell lines to examine the functional relevance of PANX1. Through stable knockdown, we evaluated cell proliferation, inflammatory cytokine transcription (IL6, TNFA, and IL1B), and extracellular ATP release, providing initial experimental support for PANX1 involvement in tumor-inflammation coupling. Collectively, our work links inflammation-informed big-data discovery to multi-omics characterization and experimental validation, nominating PANX1 as a biomarker/functional candidate at the tumor-inflammation interface in LUAD that requires further mechanistic and translational validation.

## 2 Methods

### 2.1 Patient cohorts and data preprocessing

Transcriptomic profiles and corresponding clinical annotations for lung adenocarcinoma (LUAD) were obtained from publicly available datasets in The Cancer Genome Atlas (TCGA) and Gene Expression Omnibus (GEO) (Weinstein et al., 2013; Barrett et al., 2011). The TCGA-LUAD cohort served as the primary discovery and model-development dataset, comprising 535 tumor samples and 59 adjacent normal lung tissues. Patients with available survival information were retained for prognostic modeling and downstream analyses. To evaluate the external performance and generalizability of the prognostic model, a total of 13 independent LUAD cohorts were retrieved from GEO, including GSE19188 (n = 91), GSE26939 (n = 116), GSE29016 (n = 39), GSE31210 (n = 226), GSE3141 (n = 111), GSE37745 (n = 106), GSE41271 (n = 182), GSE50081 (n = 127), GSE63459 (n = 33), GSE68465 (n = 442), GSE68571 (n = 86), GSE72094 (n = 442), and GSE8894 (n = 138). Survival-related endpoints were analyzed according to their availability and original cohort annotations, including OS, DSS, PFI, DFI, RFS, and DFS; these endpoints were not interpreted as interchangeable clinical outcomes. Raw expression matrices were downloaded from each database and processed in a standardized manner. For microarray datasets, probe identifiers were converted to gene symbols according to platform annotation files, and the median expression value was used when multiple probes mapped to the same gene (Bolstad et al., 2003). For RNA-seq data from TCGA, normalized FPKM values were used (Wagner et al., 2012). All expression matrices were log2-transformed when necessary and normalized within each cohort before risk-score calculation to reduce scale differences across platforms. Samples lacking survival information or essential clinical annotations were excluded prior to downstream analyses. In addition, inflammation-related genes were curated from the Molecular Signatures Database (MSigDB) Hallmark gene sets (Liberzon et al., 2015), including HALLMARK_INFLAMMATORY_RESPONSE, HALLMARK_TNFA_SIGNALING_VIA_NFKB, and HALLMARK_IL6_JAK_STAT3_SIGNALING, which served as the initial immune-inflammatory gene pool for subsequent analyses. All datasets analyzed in this study were obtained from publicly accessible repositories, and therefore no additional ethical approval was required.

### 2.2 Identification of differentially expressed and prognostic immune-inflammatory genes

Using the TCGA-LUAD cohort, we compared gene expression profiles between LUAD tumors and matched adjacent normal lung tissues. Expression data were processed and analyzed for differential expression with the R packages limma and edgeR (Ritchie et al., 2015; Robinson et al., 2010). Genes meeting |log2FC| ≥ 1 together with P < 0.05 were classified as differentially expressed genes (DEGs). To determine survival relevance, we performed univariate Cox proportional hazards modeling with the survival R package using overall survival (OS) as the endpoint. Because this step was designed as an exploratory screening filter, genes with nominal P < 0.05 in the Cox analysis were considered OS-associated and carried forward as prognostic candidates. Finally, genes simultaneously belonging to the immune-inflammatory gene pool, the DEG set, and the OS-associated set were defined as immune-inflammatory prognostic candidates for downstream analyses. The subsequent machine-learning prioritization, multi-cohort validation, and functional experiments were used to further reduce the likelihood that candidates reflected screening-stage false positives.

### 2.3 Functional enrichment analysis

Functional characterization of candidate genes was conducted through Gene Ontology (GO) and KEGG pathway enrichment using clusterProfiler (Yu et al., 2012), with gene identifiers mapped via org.Hs.eg.db. GO enrichment was evaluated across the biological process (BP), molecular function (MF), and cellular component (CC) domains, while KEGG analysis was used to highlight pathways potentially implicated in LUAD-related biology. Enrichment outputs were summarized and visualized with ggplot2 and enrichplot. Terms/pathways with adjusted P < 0.05 were considered significant.

### 2.4 Machine learning-based feature selection

To further shrink the feature space and prioritize recurrent immune-inflammatory signals, we implemented a multi-algorithm machine-learning workflow in the TCGA-LUAD cohort. For this feature-prioritization step, LUAD tumor versus adjacent-normal status was used as the supervised classification endpoint, allowing the models to identify immune-inflammatory genes that contributed to tumor-associated discrimination. Samples were randomly split into equal training and testing sets (1:1) using createDataPartition in the caret R package. A panel of supervised learners was fitted through the caret train interface, including support vector machine (SVM), gradient boosting machine (GBM), generalized linear model (GLM), logistic regression, naive Bayes, k-nearest neighbor (KNN), random forest (RF), partial least squares (PLS), elastic net, and stepwise linear discriminant analysis (stepLDA). Hyperparameters were tuned within the caret resampling framework where applicable, and model performance was evaluated in the held-out testing subset. Model interpretation and performance assessment were carried out with DALEX, which was used to build explainers and summarize predictive behavior across models. Model explainers were constructed using the explain function, and prediction accuracy was evaluated on the testing set using the predict function. Receiver operating characteristic (ROC) curves were generated to assess classification performance. To assess model prediction behavior, residual distribution analyses were performed. Reverse cumulative distribution plots and residual boxplots were used to evaluate prediction error distributions across models. Feature importance was quantified using the variable_importance function in DALEX, based on a root mean square error (RMSE)-derived loss function. Genes recurrently ranked among the top contributors across models were retained as core immune-inflammatory features for downstream prognostic modeling.

### 2.5 Consensus prognostic model construction

To derive a candidate prognostic signature, we performed consensus survival modeling using the ten core immune-inflammatory genes prioritized by machine learning. Several Cox-based modeling strategies were explored, including LASSO, Elastic Net, Ridge regression, stepwise Cox, and CoxBoost approaches. Penalized Cox models were implemented with the glmnet package, and optimal regularization parameters were selected via tenfold cross-validation (Si et al., 2011). Stepwise Cox analysis was conducted using the survival and MASS packages, whereas the CoxBoost framework was fitted using the corresponding R implementation with tuned penalty and step settings. For each model, individual risk scores were computed as weighted sums of gene expression values according to model-derived coefficients, using the formula: risk score = Σ(βi × expressioni), where βi represents the coefficient of gene i in the selected model. Predictive performance was assessed using time-dependent ROC analysis with timeROC where applicable (Blanche et al., 2013), and cross-model performance was summarized as AUC heatmaps across cohorts and available survival endpoints using ComplexHeatmap (Gu et al., 2016). The final model was chosen based on the highest mean AUC across the evaluated cohorts/endpoints, while recognizing that the mean AUC was modest and varied across settings; the Elastic_net_0.2 model was selected, and its gene coefficients are shown in Figure 3B. Patients were stratified into high- and low-risk groups using the median risk score as the threshold. Survival differences were examined with Kaplan-Meier analysis implemented in the survival and survminer packages, with significance evaluated by the log-rank test (Rich et al., 2010). Hazard ratios (HRs) were further estimated using Cox proportional hazards regression.

### 2.6 External validation of the prognostic signature

To assess the external performance and generalizability of the proposed prognostic model, we performed validation in 13 independent LUAD cohorts obtained from the GEO repository. For each external dataset, individual risk scores were computed using the coefficients derived from the optimal consensus model established in the TCGA training cohort. Patients were then categorized into high- and low-risk groups within each cohort based on the median risk score threshold. Survival outcomes were evaluated using Kaplan-Meier analysis implemented with the survival and survminer R packages, and intergroup differences were examined using the log-rank test. Because different cohorts provided different survival-related endpoints, OS, DSS, PFI, DFI, RFS, and DFS were analyzed and reported according to the endpoint available for each cohort. For transparency, the AUC and forest-plot summaries were used to present cross-cohort and cross-endpoint performance, whereas the main Kaplan-Meier figure was restricted to TCGA-LUAD and OS cohorts with statistically significant survival differences (log-rank P < 0.05); OS cohorts without statistically significant separation were not displayed in the main Kaplan-Meier panel.

### 2.7 Clinical relevance of PANX1 in LUAD

PANX1 expression was evaluated at both protein and transcriptomic levels in LUAD. Protein abundance data were obtained from the CPTAC-LUAD proteomic cohort, and samples with missing values were excluded prior to analysis (Gillette et al., 2020). Differences in PANX1 protein expression between tumor and normal tissues were assessed using the Wilcoxon rank-sum test (Nahm, 2016). For transcriptomic analyses, TCGA-LUAD RNA-seq data were used. Patients were stratified by pathological stage (Stage I–II vs. Stage III–IV), and expression differences were evaluated using the Wilcoxon rank-sum test. Molecular subtype labels (LUAD1–LUAD6) were retrieved from publicly available TCGA subtype annotation resources, and differences in gene expression across subtypes were evaluated using the Kruskal–Wallis test. Immune subtype classifications (C1, C2, C3, C4, and C6) were assigned according to the TCGA immune subtype framework. Samples were further divided into PANX1-high and PANX1-low groups using the median expression level as the cutoff. Variations in immune subtype composition between the two groups were assessed with the chi-square test.

### 2.8 Genomic alterations associated with PANX1 in LUAD

Somatic copy-number alteration (SCNA) profiles of TCGA-LUAD were summarized using GISTIC2-derived genome-wide copy-number calls, and genome-level amplification/deletion peaks were visualized by chromosome-wise GISTIC scores (Mermel et al., 2011). To characterize genome-wide copy-number burden in the context of PANX1 expression, samples were grouped into quartiles (Q1–Q4) according to PANX1 levels and compared for fraction of genome altered (FGA), as well as fractions gained (FGG) and lost (FGL). Associations between PANX1 expression and gene-level copy-number categories (−2 deep deletion, −1 shallow deletion, 0 diploid, +1 gain, +2 amplification) were evaluated, with differences across states tested using the Kruskal–Wallis test. To explore mutation-related effects, the relationship between somatic mutations and PANX1 expression was assessed using permutation-based independence testing implemented via the independence_test function in the coin package. Genes with a mutation frequency greater than 10% and permutation P < 0.01 were retained for downstream visualization. The overall mutation landscape of PANX1 in LUAD was further summarized and illustrated using the maftools package (Mayakonda et al., 2018).

### 2.9 PANX1-associated immune microenvironment features in LUAD

TCGA-LUAD samples were stratified into PANX1-high and PANX1-low groups based on the median expression level of PANX1. For immune cell deconvolution, CIBERSORT was applied to the bulk transcriptomic profiles using the LM22 signature matrix (22 immune cell subsets); the estimated cell fractions were summarized and visualized as stacked bar plots to compare the overall immune composition between PANX1 expression groups (ggplot2 and ggalluvial) (Newman et al., 2015; Chen et al., 2018). In addition, immune infiltration scores were estimated using an ssGSEA approach implemented in the GSVA package, based on a curated panel of 24 immune cell gene signatures (including aDC, B cells, CD8 T cells, cytotoxic cells, DC, eosinophils, iDC, macrophages, mast cells, neutrophils, NK subsets, pDC, T-cell subsets, and Treg). Associations between PANX1 expression and immune infiltration metrics were evaluated using Spearman correlation, and the resulting correlation matrix was visualized as a heatmap using ggplot2 (Hänzelmann et al., 2013). To increase robustness, differences in tumor microenvironment components between PANX1-high and PANX1-low groups were further assessed using seven independent algorithms, and group-wise comparisons were performed using the non-parametric Wilcoxon rank-sum test; significantly different immune features were displayed in a heatmap with samples ordered by PANX1 expression from low to high. Where applicable, Spearman correlation between PANX1 expression and TIP (cancer immunity cycle) scores as well as autocorrelation among TIP scores were visualized using the “linkET” package (Chen and Mellman, 2013).

### 2.10 Cell lines and cell culture

The human lung adenocarcinoma cell line A549 (RRID:CVCL_0023), non-small cell lung cancer cell line NCI-H1299 (RRID:CVCL_0060), immortalized human bronchial epithelial cell line BEAS-2B (RRID:CVCL_0168), and human embryonic kidney cell line HEK293 (RRID:CVCL_0045) were obtained from the American Type Culture Collection (ATCC, Manassas, VA, USA). A549 and NCI-H1299 cells were cultured in RPMI-1640 medium (Gibco, Thermo Fisher Scientific) supplemented with 10% fetal bovine serum (FBS) and 1% penicillin–streptomycin. BEAS-2B and HEK293 cells were maintained in DMEM (Gibco) containing the same supplements. All cultures were incubated at 37 °C under 5% CO_2_ in a humidified incubator and were routinely screened to ensure the absence of *mycoplasma* contamination before downstream experiments.

### 2.11 Stable PANX1 knockdown construction

Stable PANX1 knockdown cell lines were generated using a lentiviral shRNA delivery system. shRNA sequences targeting PANX1, together with a non-targeting control (shNC), were inserted into the pLKO.1-puro vector. Lentiviral particles were produced in HEK293 packaging cells by co-transfecting the shRNA plasmids with psPAX2 and pMD2. G using Lipofectamine 3,000 (Thermo Fisher Scientific) following the manufacturer’s protocol. Viral supernatants were harvested at 48 and 72 h after transfection, passed through a 0.45 µm filter, and subsequently used to infect A549 and NCI-H1299 cells in the presence of polybrene (8 μg/mL). Infected cells were selected with puromycin after 48 h to establish stable knockdown lines. The efficiency of PANX1 silencing was confirmed by quantitative real-time PCR before functional assays were conducted.

### 2.12 Quantitative real-time PCR

Total RNA was isolated from cultured cells using TRIzol reagent (Invitrogen, Thermo Fisher Scientific) in accordance with the manufacturer’s protocol. RNA quantity and purity were assessed with a NanoDrop spectrophotometer. For cDNA synthesis, 1 μg of total RNA was reverse-transcribed using the PrimeScript RT reagent kit (Takara, Japan). Quantitative real-time PCR was carried out using TB Green Premix Ex Taq II (Takara) on a QuantStudio real-time PCR system (Applied Biosystems). Cycling parameters followed the recommended conditions provided by the manufacturer. Relative transcript levels were calculated using the 2^−ΔΔCt^ method, with GAPDH used as the endogenous control. All assays were conducted in triplicate. The primer sequences used in this study are listed below:

GAPDH-F: GAC​AGT​CAG​CCG​CAT​CTT​CT
GAPDH-R: GCG​CCC​AAT​ACG​ACC​AAA​TC
PANX1-F: AGAGGCGCGAATCCGAGT
PANX1-R: ACC​AAT​CGA​GAT​CTC​CTG​CG
IL6-F: CCT​TCG​GTC​CAG​TTG​CCT​TCT
IL6-R: TCT​GAG​GTG​CCC​ATG​CTA​CA
TNFA-F: CTG​GGC​AGG​TCT​ACT​TTG​GG
TNFA-R: CTG​GAG​GCC​CCA​GTT​TGA​AT
IL1B-F: CCA​AAC​CTC​TTC​GAG​GCA​CA
IL1B-R: AGC​CAT​CAT​TTC​ACT​GGC​GA

### 2.13 Cell viability assay (CCK-8)

Cell viability was evaluated using the Cell Counting Kit-8 (CCK-8; Dojindo, Japan) following the manufacturer’s guidelines. Cells were plated in 96-well plates at a density of 2 × 10^3^ cells per well and allowed to adhere overnight. At the designated time points (0, 24, 48, 72, and 96 h), 10 μL of CCK-8 solution was added to each well containing 100 μL of culture medium and incubated at 37 °C for 1–2 h. Absorbance was then recorded at 450 nm using a microplate reader, and cell viability was represented as OD450 values. Each condition included at least three technical replicates, and all experiments were independently performed three times.

### 2.14 Extracellular ATP release assay

Extracellular ATP levels were quantified using a luminescence-based ATP detection kit (e.g., ATP Determination Kit, Thermo Fisher Scientific) following the manufacturer’s instructions. Briefly, stable PANX1 knockdown cells and corresponding controls were plated in 24-well plates and cultured until reaching approximately 70%–80% confluence. Cells were rinsed once with pre-warmed PBS and then incubated in phenol red–free, serum-free medium or HBSS at 37 °C for 30 min. Supernatants were carefully collected without disturbing the cell layer and centrifuged at 500 *g* for 5 min at 4 °C to remove debris. The clarified supernatants were transferred to white 96-well plates, mixed with the luciferase/luciferin reagent, and luminescence was measured using a microplate luminometer. ATP concentrations were determined based on a standard curve generated with kit-provided ATP standards. Extracellular ATP levels were normalized to the total cellular protein content from corresponding wells and expressed as fold change relative to the control group. All assays were performed in triplicate, and each experiment was independently repeated at least three times.

### 2.15 Statistical analysis

All statistical analyses were conducted using R software. Continuous variables were evaluated using the Wilcoxon rank-sum test for comparisons between two groups and the Kruskal–Wallis test for analyses involving more than two groups. Survival analyses were conducted using the Kaplan-Meier method, and differences between groups were evaluated by the log-rank test. Hazard ratios (HRs) and 95% confidence intervals (CIs) were calculated using Cox proportional hazards regression models. Correlation analyses were performed using Spearman’s rank correlation coefficient. For multiple testing scenarios, adjusted P values were calculated where applicable. All statistical tests were two-sided, and a P value <0.05 was considered statistically significant.

## 3 Results

### 3.1 Identification of immune-inflammatory prognostic genes in LUAD

**FIGURE 1:**
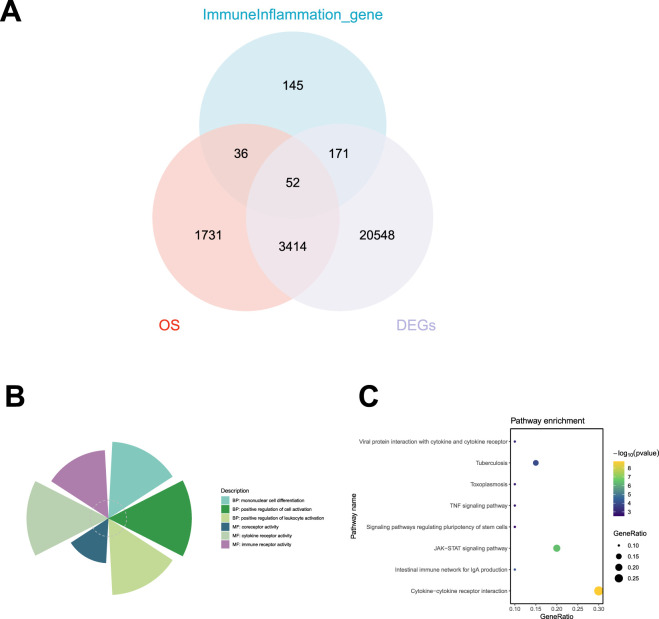
Identification of immune-inflammatory prognostic genes in LUAD. **(A)** Venn diagram showing the overlap among immune-inflammatory genes, differentially expressed genes, and survival-associated genes in the TCGA-LUAD cohort. Fifty-two genes were identified at the intersection of all three sets. **(B)** GO enrichment analysis of the intersected genes, highlighting immune activation and receptor-related terms. **(C)** KEGG pathway enrichment analysis of the intersected genes, including cytokine-cytokine receptor interaction, JAK-STAT signaling, TNF signaling, and related immune pathways.

To identify immune-inflammatory genes with potential prognostic relevance in LUAD, we integrated three gene sets derived from the TCGA-LUAD cohort: differentially expressed genes, OS-associated genes, and curated immune-inflammatory genes. Venn analysis revealed 52 genes shared among immune-inflammatory genes, DEGs, and OS-associated genes (Figure 1A). These overlapping genes concurrently displayed tumor-specific dysregulation, survival relevance, and inflammatory characteristics, indicating a close relationship with LUAD biology. To explore their functional roles, we conducted GO and KEGG enrichment analyses. GO results highlighted enrichment in immune activation and inflammatory processes, including mononuclear cell differentiation, regulation of cell activation, leukocyte activation, coreceptor activity, cytokine receptor activity, and immune receptor activity (Figure 1B). Consistently, KEGG analysis showed overrepresentation of immune- and inflammation-related pathways, such as cytokine-cytokine receptor interaction, JAK-STAT signaling, TNF signaling, intestinal immune network for IgA production, and viral protein interaction with cytokine and cytokine receptor (Figure 1C). These findings suggest that the identified genes represent a biologically grounded candidate set for subsequent machine learning-based feature selection.

### 3.2 Machine learning-based identification of core immune-inflammatory genes

**FIGURE 2:**
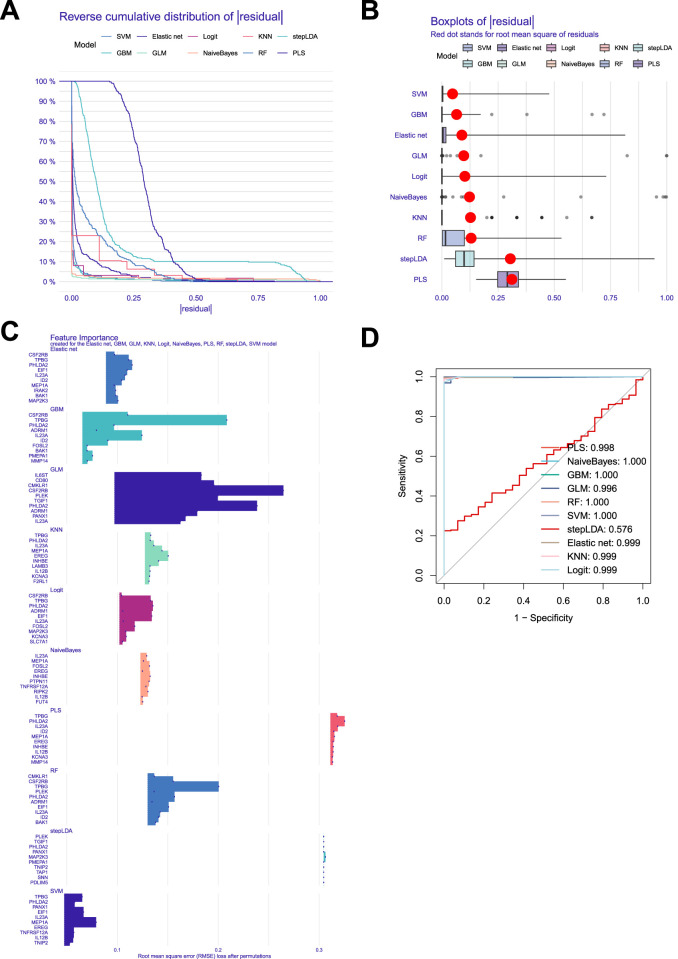
Machine learning-based identification of core immune-inflammatory genes. **(A)** Reverse cumulative residual distribution plots illustrating the concentration of prediction errors across models in the tumor-versus-normal feature-prioritization task. **(B)** Residual boxplots showing the variability and dispersion of prediction errors across machine learning models. **(C)** Variable importance ranking based on RMSE-derived loss, highlighting recurrently prioritized genes across algorithms. **(D)** ROC curves demonstrating the classification performance of machine learning models in the testing cohort.

To refine the immune-inflammatory candidate genes and improve model reproducibility, a multi-algorithm machine learning framework was applied to the TCGA-LUAD cohort. The dataset was randomly partitioned into training and testing subsets, and multiple models were trained to evaluate predictive behavior and feature importance across independent learners. Residual diagnostics indicated broadly similar model behavior in the held-out testing subset. Reverse cumulative residual plots showed that residuals were predominantly centered near zero across models, indicating limited deviation between predicted and reference values (Figure 2A). Residual boxplots further demonstrated compact interquartile ranges with limited extreme deviations for most models (Figure 2B). Feature attribution analysis revealed notable cross-model concordance. Variable importance ranking based on RMSE-derived loss repeatedly prioritized a subset of genes across different algorithms (Figure 2C). This convergence suggests that the identified features represent recurrent predictive signals rather than model-specific artifacts. ROC analysis further supported these observations, with most models showing favorable classification performance for the tumor-versus-normal prioritization task (Figure 2D). Based on the consensus importance ranking across machine learning models, 10 core immune-inflammatory genes were identified, including IL23A, PHLDA2, TPBG, CSF2RB, MEP1A, ID2, ADRM1, EIF1, FOSL2, and PANX1. These genes were retained for subsequent consensus prognostic modeling.

### 3.3 Consensus prognostic modeling based on immune-inflammatory genes

**FIGURE 3:**
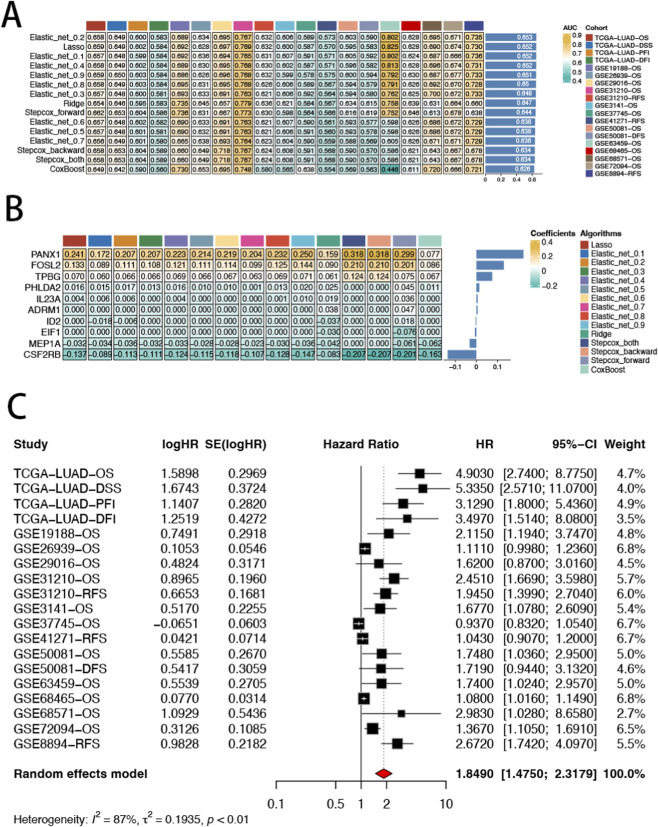
Consensus prognostic modeling based on core immune-inflammatory genes. **(A)** Heatmap comparing summary AUC values across TCGA/GEO cohorts and available survival endpoints for different survival modeling algorithms. **(B)** Coefficient heatmap showing gene contributions across modeling frameworks; the Elastic_net_0.2 coefficients were used to calculate the final risk score. **(C)** Forest plot summarizing hazard ratios for available survival-related endpoints across TCGA and GEO cohorts based on the optimal prognostic model.

To establish a candidate prognostic signature, consensus survival modeling was performed using the 10 core immune-inflammatory genes identified in the previous step. Multiple Cox regression frameworks were evaluated to compare predictive performance and model behavior. AUC analysis demonstrated variation in predictive performance across algorithms and validation settings. Comparative AUC heatmaps revealed that penalized regression frameworks generally performed well relative to the other evaluated approaches across TCGA/GEO cohorts and available survival endpoints (Figure 3A). Among them, the Elastic_net_0.2 model achieved the highest mean AUC (0.653) across the evaluated cohorts and endpoints and was therefore selected as the final prognostic model; however, this value indicates moderate discrimination rather than strong predictive accuracy. Coefficient heatmaps further illustrated the contribution of each gene across different modeling strategies (Figure 3B). The final risk score was calculated as the weighted sum of the expression values of the 10 genes using the Elastic_net_0.2 coefficients shown in Figure 3B. Using the selected optimal model, an integrated risk score was generated for each patient. A cross-cohort forest plot summarizing available survival-related endpoints showed that the high-risk group was generally associated with poorer outcomes than the low-risk group (random-effects HR = 1.8490, 95% CI 1.4750–2.3179; Figure 3C), although heterogeneity across endpoints and cohorts was high (I^2 = 87%). These findings indicate that the consensus modeling framework captures prognostic signals for subsequent multi-cohort validation, but the magnitude and consistency of performance should be interpreted cautiously.

### 3.4 External validation of the immune-inflammatory prognostic signature

**FIGURE 4:**
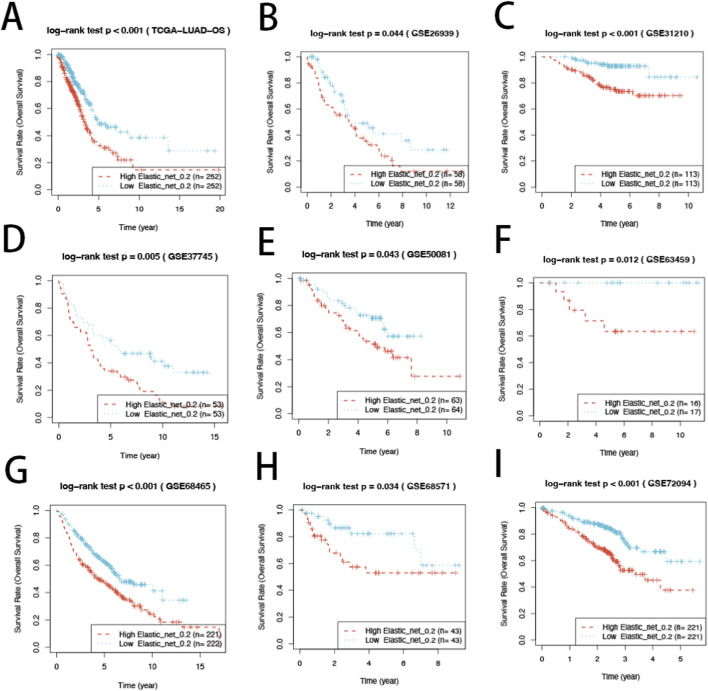
Kaplan-Meier validation of the immune-inflammatory prognostic signature across TCGA and GEO cohorts. Kaplan-Meier survival curves are shown for TCGA-LUAD and independent GEO datasets with available OS information that showed statistically significant survival differences. Risk scores were calculated using the TCGA-derived prognostic model, and patients were stratified into high- and low-risk groups based on the median cutoff. OS cohorts without statistically significant log-rank separation were not displayed in this main visualization; cross-cohort and cross-endpoint performance was summarized separately in the AUC and forest-plot analyses. Panels **(A-I)** correspond to TCGA-LUAD-OS, GSE26939, GSE31210, GSE37745, GSE50081, GSE63459, GSE68465, GSE68571, and GSE72094, respectively.

To further assess the external performance of the immune-inflammatory prognostic signature, we conducted validation using TCGA-LUAD and 13 independent LUAD cohorts from the GEO database. Risk scores for each dataset were computed based on coefficients derived from the optimal consensus model, and patients were divided into high- and low-risk groups using the median cutoff. Kaplan-Meier analyses demonstrated survival differences across TCGA-LUAD and multiple independent GEO cohorts (Figure 4). Among datasets with available overall survival data, individuals in the high-risk category generally showed worse outcomes than those in the low-risk group, but the strength and statistical significance of separation varied by cohort. For clarity of the main visualization, TCGA-LUAD and OS cohorts exhibiting statistically significant survival differences (log-rank P < 0.05) were included in Figure 4, whereas OS cohorts without statistically significant survival separation were not shown in this main Kaplan-Meier panel and additional survival-related endpoints were summarized separately according to their original cohort definitions. Collectively, these results indicate that the immune-inflammatory signature retains moderate prognostic performance across diverse external populations, while endpoint heterogeneity and cohort-specific variability should be considered when interpreting cross-cohort validation.

### 3.5 Clinical and molecular expression landscape of PANX1 in LUAD

**FIGURE 5:**
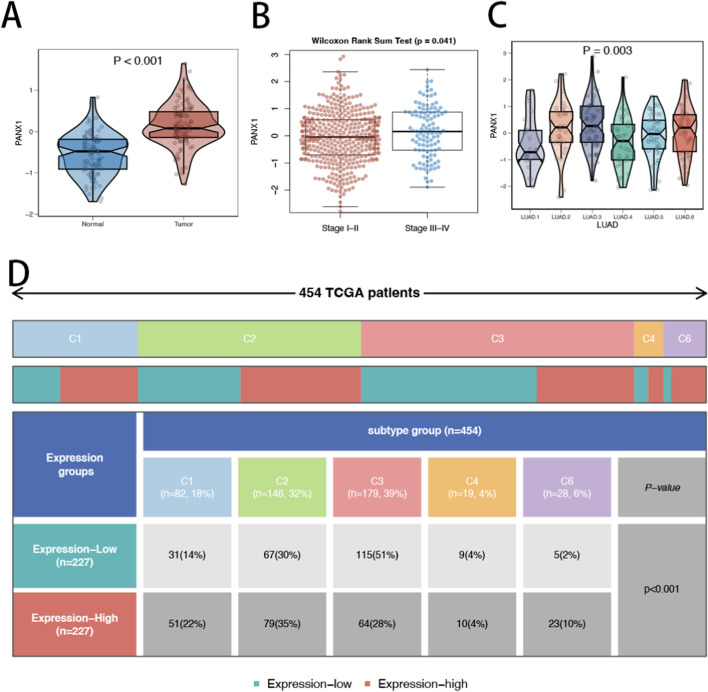
Clinical and molecular expression characteristics of PANX1 in LUAD. **(A)** PANX1 protein expression in the CPTAC-LUAD cohort comparing tumor and normal tissues. **(B)** PANX1 mRNA expression in TCGA-LUAD stratified by pathological stage (Stage I–II vs. Stage III–IV). **(C)** PANX1 expression across LUAD molecular subtypes (LUAD1–LUAD6). **(D)** Distribution of TCGA immune subtypes (C1, C2, C3, C4, and C6) between PANX1-high and PANX1-low groups defined by the median cutoff.

PANX1 was selected for single-gene characterization based on an integrative assessment rather than a single quantitative ranking metric. Specifically, PANX1 belonged to the 10 recurrently prioritized immune-inflammatory genes, showed a visible contribution in the selected Elastic_net_0.2 model, recurred across model construction and downstream molecular/immune analyses, and has known biological relevance to ATP-mediated intercellular inflammatory signaling. Thus, PANX1 was treated as a focused biomarker/functional candidate requiring further validation, not as a proven central regulator. To further clarify its clinical and biological context in LUAD, we systematically evaluated PANX1 expression across proteomic, clinicopathological, and molecular stratifications. At the proteomic level, PANX1 protein abundance was significantly elevated in LUAD tumors compared with normal tissues in the CPTAC-LUAD cohort (Figure 5A, Wilcoxon P < 0.001), supporting tumor-associated upregulation at the protein level. Consistently, in the TCGA-LUAD transcriptomic cohort, PANX1 expression was significantly higher in advanced-stage tumors (Stage III-IV) than in early-stage tumors (Stage I-II) (Figure 5B, Wilcoxon P = 0.041), suggesting an association with advanced-stage disease. PANX1 expression also exhibited significant heterogeneity across LUAD molecular subtypes (LUAD1-LUAD6) (Figure 5C, Kruskal-Wallis P = 0.003), indicating subtype-dependent expression patterns. In addition, PANX1 expression was linked to tumor immune phenotypes: among 454 TCGA-LUAD patients dichotomized by the median PANX1 level (PANX1-high, n = 227; PANX1-low, n = 227), immune subtype composition differed significantly between groups (Figure 5D, chi-square P < 0.001). The PANX1-low group was enriched for the C3 subtype (51% vs. 28%), whereas the PANX1-high group showed higher proportions of C1 (22% vs. 14%), C2 (35% vs. 30%), and particularly C6 (10% vs. 2%). Collectively, these findings demonstrate that PANX1 upregulation in LUAD is associated with tumor progression and distinct molecular and immune subtype distributions.

### 3.6 PANX1 is embedded in a distinct genomic alteration context in LUAD

**FIGURE 6:**
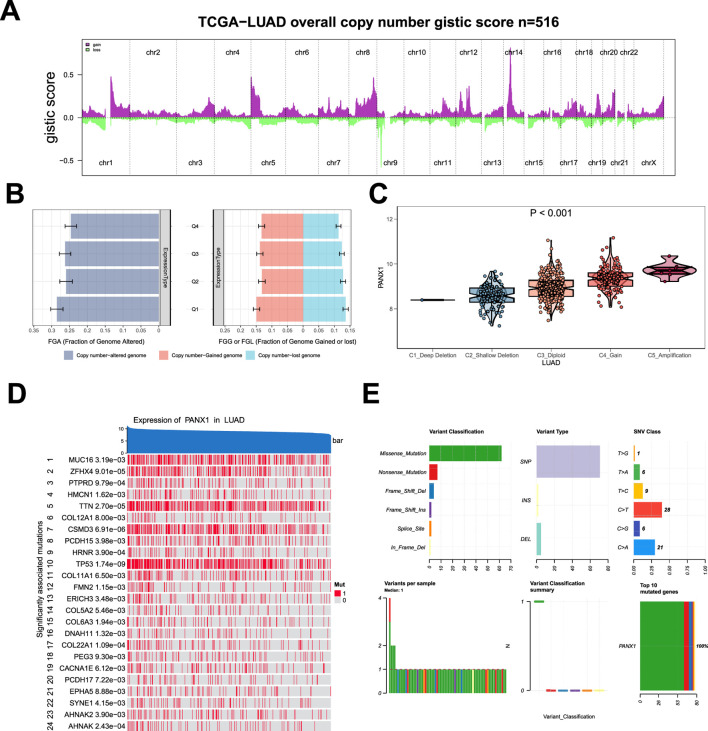
Genomic alterations associated with PANX1 in LUAD. **(A)** Genome-wide GISTIC2 copy-number scores across TCGA-LUAD samples, showing amplification and deletion peaks across chromosomes. **(B)** Global SCNA burden stratified by PANX1 expression levels, including fractions of genome altered, gained, and lost. **(C)** PANX1 expression across discrete copy-number states (deep deletion, shallow deletion, diploid, gain, amplification). **(D)** Mutation–expression associations between PANX1 and recurrently mutated genes identified by permutation testing. **(E)** PANX1 mutation summary in Pan-cancer, including variant classification, variant type, and SNV class distribution.

Genome-wide SCNA profiling in TCGA-LUAD (n = 516) revealed widespread chromosomal gains and losses across the cohort, with recurrent amplification and deletion signals captured by GISTIC scores along the genome (Figure 6A). When stratifying tumors by PANX1 expression quartiles, global SCNA burden (FGA) and the proportions of copy-number gains/losses (FGG/FGL) showed evident differences across expression strata, indicating that PANX1 expression is associated with broader genomic alteration patterns at the cohort level (Figure 6B). At the gene level, PANX1 expression differed significantly across discrete copy-number states, with expression increasing stepwise from deletion/diploid states toward gain/amplification categories (Figure 6C, Kruskal-Wallis P < 0.001), supporting a copy-number-expression dosage relationship. In addition, permutation-based mutation-expression association analysis identified multiple recurrently mutated genes whose mutation status was significantly linked to PANX1 expression (e.g., MUC16, ZFHX4, PTPRD, HMCN1, TTN, COL12A1, CSMD3, PCDH15, HRNR, and TP53; Figure 6D; visualized under the criteria of mutation frequency >10% and permutation P < 0.01). To extend these observations beyond a single cancer type, we next examined the pan-cancer mutation landscape of PANX1. Across cancer types, PANX1 alterations were predominantly missense mutations and were largely composed of single-nucleotide variants, with C>A and C>T substitutions representing the most frequent SNV classes (Figure 6E). These results suggest that PANX1 expression is associated with distinct genomic contexts in LUAD, although the direction and causality of these relationships cannot be inferred from the present analyses.

### 3.7 PANX1 is associated with immune infiltration heterogeneity in LUAD

**FIGURE 7:**
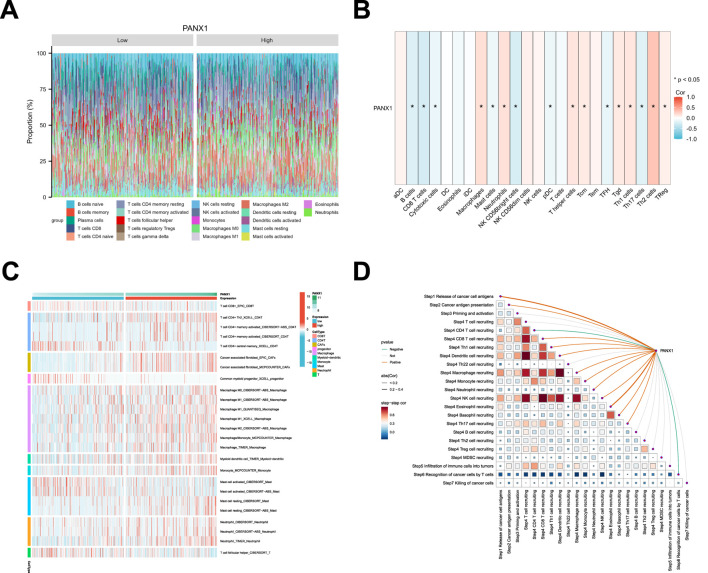
PANX1-associated inferred immune infiltration and immune functional features in LUAD. **(A)** Stacked bar plot summarizing immune cell fractions estimated by CIBERSORT (LM22) in PANX1-high versus PANX1-low tumors (median cutoff). **(B)** Heatmap of Spearman correlations between PANX1 expression and ssGSEA-derived immune cell infiltration scores (24 immune cell signatures). **(C)** Heatmap summarizing significantly different microenvironment components between PANX1-high and PANX1-low groups across seven algorithms; samples are ordered from low to high PANX1 expression. **(D)** linkET visualization of Spearman correlations between PANX1 expression and TIP (cancer immunity cycle) scores and the autocorrelation structure among TIP scores.

Immune deconvolution revealed that PANX1 expression was associated with inferred tumor immune composition in LUAD. Using CIBERSORT (LM22), stacked bar plots demonstrated an overall shift in the relative abundance of multiple immune cell subsets between PANX1-high and PANX1-low tumors (Figure 7A), supporting group-level differences in inferred immune infiltration composition. Consistently, ssGSEA-based immune scoring showed broad associations between PANX1 and immune cell infiltration signatures: Spearman correlation analysis indicated that PANX1 expression correlated with multiple immune cell scores across the 24 immune cell types, highlighting coordinated changes in adaptive and innate immune compartments (Figure 7B). When immune microenvironment components were re-estimated using seven independent algorithms, Wilcoxon testing identified a panel of immune features that differed significantly between PANX1-high and PANX1-low groups, and these features displayed expression-ordered patterns when samples were arranged from low to high PANX1 expression (Figure 7C), indicating that PANX1 captures a reproducible microenvironment-associated gradient. In addition, PANX1 expression showed measurable associations with cancer immunity cycle (TIP) activities, and the linkET-based visualization illustrated both gene-TIP correlations and the internal correlation structure among TIP steps (Figure 7D). Together, these analyses support that PANX1 is linked to broad inferred immune infiltration and immune functional states in LUAD, but they do not establish direct regulation of immune-cell recruitment.

### 3.8 PANX1 knockdown impairs proliferative capacity in LUAD cells

**FIGURE 8:**
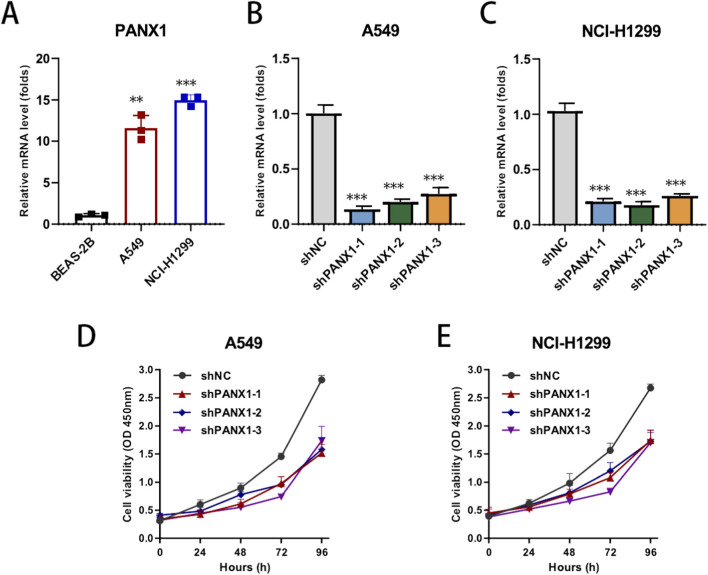
PANX1 is upregulated in LUAD cell lines, and PANX1 knockdown suppresses cell growth. **(A)** qRT–PCR analysis of PANX1 mRNA expression in BEAS-2B, A549, and NCI-H1299 cells. **(B,C)** qRT–PCR validation of PANX1 knockdown efficiency in A549 **(B)** and NCI-H1299 **(C)** cells transduced with shNC or three independent shPANX1 constructs (shPANX1-1/-2/-3). **(D,E)** CCK-8 growth curves in A549 **(D)** and NCI-H1299 **(E)** cells following PANX1 knockdown. Data are shown as mean ± SD (error bars). **P < 0.01, ***P < 0.001.

To validate PANX1 expression in LUAD cells, qRT–PCR showed that PANX1 mRNA levels were markedly higher in A549 and NCI-H1299 cells than in BEAS-2B cells (Figure 8A; **P < 0.01 and ***P < 0.001, respectively). We next generated stable PANX1 knockdown models using three independent shRNA constructs. All three shPANX1 constructs (shPANX1-1/-2/-3) substantially reduced PANX1 mRNA levels in both A549 and NCI-H1299 cells compared with the shNC control (Figures 8B,C; ***P < 0.001). Functionally, CCK-8 assays revealed that PANX1 knockdown consistently attenuated cell viability in both LUAD cell lines over time, with shPANX1 groups showing lower OD450 trajectories than shNC, particularly at later time points (Figures 8D,E). These results support that PANX1 is upregulated in LUAD cells and that reducing PANX1 expression is accompanied by impaired proliferative capacity *in vitro*.

### 3.9 PANX1 knockdown attenuates pro-inflammatory signaling in LUAD cells

**FIGURE 9:**
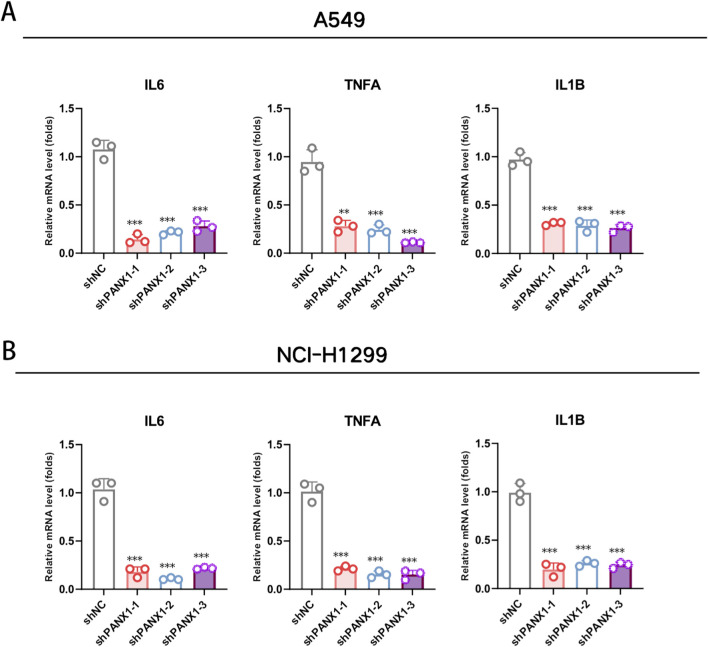
PANX1 knockdown reduces pro-inflammatory cytokine expression in LUAD cells. **(A)** qRT–PCR analysis of IL6, TNFA, and IL1B mRNA levels in A549 cells transduced with shNC or three independent shPANX1 constructs. **(B)** qRT–PCR validation of cytokine expression changes in NCI-H1299 cells. Expression levels were normalized to an internal control and presented relative to shNC. Data are shown as mean ± SD from independent experiments. **P < 0.01, ***P < 0.001 versus shNC.

To further explore whether PANX1 is linked to inflammatory signaling in LUAD cells, we quantified the expression of representative pro-inflammatory cytokines following PANX1 knockdown. qRT-PCR analysis demonstrated that silencing PANX1 markedly reduced the mRNA levels of IL6, TNFA, and IL1B in A549 cells compared with shNC controls (Figure 9A). A similar pattern was observed in NCI-H1299 cells, where PANX1 depletion consistently suppressed the expression of all three cytokines across independent shRNA constructs (Figure 9B). The concordant downregulation of multiple inflammatory mediators in two LUAD cell lines suggests that PANX1 is associated with a pro-inflammatory transcriptional state in lung cancer cells, although protein-level cytokine secretion remains to be validated.

### 3.10 PANX1 knockdown reduces extracellular ATP release in lung cancer cells

**FIGURE 10:**
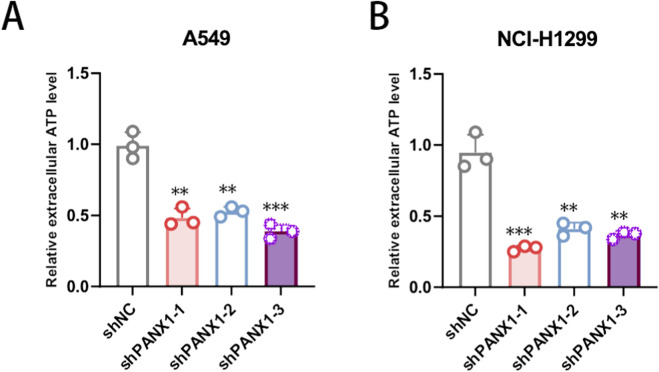
PANX1 knockdown reduces extracellular ATP release in lung cancer cells. Extracellular ATP levels were measured using a luminescence-based ATP assay in stable PANX1 knockdown A549 and NCI-H1299 cells. Cells transduced with shNC served as controls. ATP levels were normalized and presented as fold change relative to shNC. Silencing of PANX1 significantly decreased extracellular ATP release in both lung cancer cell lines. Data are presented as mean ± SD from three independent experiments. **P < 0.01, ***P < 0.001 versus shNC. Downstream purinergic signaling pathways were not directly assessed in this assay. **(A)** A549 cells. **(B)** NCI-H1299 cells.

To determine whether PANX1 is linked to extracellular ATP release in lung cancer cells, we quantified extracellular ATP levels following stable PANX1 knockdown. A luminescence-based ATP assay revealed that silencing PANX1 significantly reduced extracellular ATP release in both A549 and NCI-H1299 cells compared with shNC controls (Figure 10). This reduction was repeatedly observed across independent shPANX1 constructs, indicating an association between PANX1 depletion and reduced ATP secretion. Given that extracellular ATP is a key mediator of inflammatory signaling and tumor-microenvironment communication, these findings further support PANX1 as a potential biomarker/functional candidate related to inflammatory output in lung cancer cells, while downstream purinergic signaling pathways require further investigation.

## 4 Discussion

This study integrates inflammation-informed feature selection, machine learning prioritization, multi-cohort validation, and functional experiments to identify PANX1 as an immune-inflammatory candidate regulator associated with LUAD (Liu et al., 2024). Starting from hallmark inflammatory pathways, we intersected immune-inflammatory genes with LUAD differential expression and OS-associated genes, prioritized candidates using multiple machine learning models, and derived a 10-gene core signature that showed prognostic value across TCGA-LUAD and independent GEO cohorts. PANX1 was not selected solely because it ranked first by a single quantitative metric; instead, it was chosen as a focused candidate based on an integrative assessment of model contribution, recurrence across analyses, and biological plausibility related to ATP-mediated inflammatory communication (Abdo et al., 2023).

A useful aspect of this study is its multi-cohort design, although the prognostic performance should be interpreted with appropriate caution. Prognostic signatures often perform well in a discovery cohort but fail to generalize due to platform effects and cohort heterogeneity. Here, the use of biologically constrained candidate selection, multi-algorithm prioritization, and external validation across multiple datasets reduces overfitting risk and supports that the risk score captures an inflammation-associated phenotype (Sweeney et al., 2017). However, the mean AUC was modest, and survival endpoints and cohort characteristics were heterogeneous, indicating that the signature should be viewed as a candidate risk-stratification model rather than a definitive prognostic tool. In parallel, multi-omics profiling placed PANX1 within a distinct genomic alteration context, including copy-number states and mutation-linked transcriptional patterns, suggesting PANX1-high tumors may represent a recurrent molecular pattern rather than stochastic expression variation (Mohr et al., 2024). These results should nevertheless be interpreted as associations rather than evidence that PANX1 drives genomic instability or mutation patterns.

Our immune analyses further indicated that PANX1 is associated with differences in LUAD tumor microenvironment states (Mellman et al., 2023). Across immune deconvolution and immune scoring approaches, PANX1 expression was associated with altered inferred immune infiltration composition and cancer immunity cycle activity, consistent with broad differences in the tumor immune contexture. Although bulk-based inference cannot substitute for direct cell-resolved measurements, the concordance across algorithms supports PANX1 as a candidate marker associated with inferred immune-state variation.

Importantly, experimental validation supported the computational findings. PANX1 was upregulated in LUAD cell lines compared with a normal bronchial epithelial line. Stable PANX1 knockdown reduced proliferative capacity in two LUAD cell lines and attenuated inflammatory signaling, reflected by decreased IL6, TNFA, and IL1B transcription. In addition, PANX1 depletion reduced extracellular ATP release, aligning with the known role of pannexin channels in mediating ATP efflux and suggesting a potential route by which PANX1 may couple tumor-intrinsic programs to inflammatory cues (Rusiecka et al., 2022). Together, these data support PANX1 as a biomarker/functional candidate rather than a purely correlative marker, while additional mechanistic experiments are required to establish causal downstream pathways (Huang et al., 2024).

Several limitations warrant consideration. First, while multi-cohort validation supports generalizability, differences in sample processing, clinical annotation, endpoint availability, and RNA-seq/microarray platforms across GEO datasets may introduce residual confounding, and the model showed moderate discrimination with high heterogeneity across cohorts and endpoints. Second, the initial survival screening used a nominal Cox P-value threshold as an exploratory filter; although downstream machine learning, external validation, and experimental assays were used to strengthen candidate prioritization, false-positive candidates cannot be completely excluded. Third, immune infiltration was inferred from bulk expression data and is sensitive to algorithmic assumptions; direct validation using spatial, flow-cytometric, or single-cell methods would be needed to define cell-type specificity. Fourth, mechanistic depth is limited by the use of *in vitro* loss-of-function assays; rescue or gain-of-function experiments, protein-level cytokine measurements such as ELISA, downstream P2RX/P2RY-related purinergic signaling assays, and validation in animal models or patient-derived systems would further strengthen causal interpretation and translational relevance.

## 5 Conclusion

Using a multi-cohort, machine learning-guided strategy anchored to hallmark inflammatory pathways, we derived a 10-gene immune-inflammatory signature associated with LUAD prognosis across TCGA and external GEO cohorts, with moderate predictive discrimination and notable cohort/endpoint heterogeneity. PANX1 emerged as a focused biomarker/functional candidate and was associated with genomic alteration patterns and inferred immune microenvironment differences. Functional assays further showed that PANX1 knockdown suppresses LUAD cell proliferation, reduces extracellular ATP release, and downregulates IL6, TNFA, and IL1B expression. These findings support PANX1 as a biomarker candidate and potential functional regulator of tumor-inflammation coupling in LUAD, while further mechanistic and translational validation is required before therapeutic targeting can be inferred.

## Data Availability

All datasets used in this study are publicly accessible. Transcriptomic and clinical data for TCGA-LUAD were retrieved from The Cancer Genome Atlas (https://portal.gdc.cancer.gov/). Independent validation cohorts were obtained from the Gene Expression Omnibus (GEO) repository (https://www.ncbi.nlm.nih.gov/geo/). Proteomic data were sourced from the CPTAC data portal (https://proteomics.cancer.gov/programs/cptac). Processed datasets and analysis scripts can be obtained from the corresponding author upon reasonable request.
